# Supplementary material for: Potential of Host Serum Protein Biomarkers in the Diagnosis of Tuberculous Meningitis in Children
Source: Front Pediatr. 2019 Sep 25;7:376. doi: 10.3389/fped.2019.00376 (PMC6773834; doi:10.3389/fped.2019.00376)
Supplement: Supplementary file 1 [file Table_1.DOCX]

Supplementary Material

# Supplementary Figures and Tables

## Supplementary Table

**Supplementary Table 1.** **Usefulness of all individual host biomarkers evaluated in the study in the diagnosis of TBM in children.** Median levels of host markers detected in serum samples from children with TBM or no TBM (Inter-quartile ranges in parenthesis) and accuracies in the diagnosis TBM**.** The data shown are raw, ‘unmanipulated’ values (not winsorized or standardized). Cut-off values and associated sensitivities and specificities were selected based on the Youden’s index. P-values were calculated using the Mann Whitney U test. *values shown are the absorbance and not the concentrations. #values shown are in ng/ml, values for all other host markers are in pg/ml.

| **Markers** | **Median in TBM (IQR)** | **Median in Non-TBM (IQR)** | **p-value** | **AUC (95% CI)** | **Cut-off Value** | **Sensitivity % (95% CI)** | **Specificity % (95% CI)** |
| --- | --- | --- | --- | --- | --- | --- | --- |
| VCAM-1 | 1197300.0 (952940.0-1543500.0) | 1802000.0 (1456400.0-2521350.0) | 0.000188 | 0.82 (0.70-0.94) | <1580000.0 | 78.3 (56.3-92.5) | 66.7 (44.7-84.4) |
| MCP-1/CCL2 | 244.3 (165.5-390.9) | 512.3 (319.7-994.8) | 0.000262 | 0.81 (0.69-0.93) | <327.3 | 73.9 (51.6-89.7) | 75.0 (53.3-90.2) |
| IL-4 | 82.7 (7.42-99.6) | 136.8 (99.6-191.3) | 0.001147 | 0.78 (0.65-0.91) | <116.7 | 78.3 (56.3-92.5) | 62.5 (40.6-81.2) |
| TNF-α | 4.8 (0.0-11.4) | 23.3 (15.7-31.9) | 0.001457 | 0.77 (0.62-0.91) | <12.9 | 78.3 (56.3-92.5) | 79.2 (57.9-92.9) |
| MIP-1β/ CCL4 | 219.0 (158.1-296.8) | 401.4 (275.7-667.2) | 0.002148 | 0.76 (0.62-0.90) | <334.3 | 78.3 (56.3-92.5) | 66.7 (44.7-84.4) |
| #Adipsin (CFD) | 1950.4 (1611.1-2319.1) | 2917.4 (2493.4-3938.5) | 0.004065 | 0.75 (0.59-0.90) | <2393.0 | 78.3 (56.3-92.5) | 79.2 (57.9-92.9) |
| #SAP | 331539.9 (261542.1-655100.2) | 167660.5 (88309.6-286067.7) | 0.005664 | 0.74 (0.59-0.89) | >257478.0 | 78.3 (56.3-92.5) | 70.8 (48.9-87.4) |
| #CC5 | 52307.0 (44989.9-59967.4) | 38538.0 (28210.6-47089.8) | 0.006660 | 0.73 (0.58-0.88) | >46742.0 | 69.6 (47.1-86.8) | 75.0 (53.3-90.2) |
| #CFH | 415846.5 (363515.9-470137.5) | 314294.0 (261691.8-412727.7) | 0.009719 | 0.72 (0.57-0.87) | >350185.0 | 87.0 (66.4-97.2) | 66.7 (44.7-84.4) |
| G-CSF | 14.0 (0.0-117.6) | 147.6 (25.1-463.4) | 0.010573 | 0.72 (0.57-0.86) | <76.0 | 65.2 (42.7-83.6) | 70.8 (48.9-87.4) |
| #Apo CIII | 151289.3 (130100.8-181642.4) | 95825.1 (63481.7-161543.8) | 0.014822 | 0.71 (0.55-0.87) | >114926.0 | 87.0 (66.4-97.2) | 62.5 (40.6-81.2) |
| IL-10 | 0.0 (0.0-4.1) | 8.1 (0.0-21.2) | 0.011193 | 0.70 (0.56-0.85) | <7.0 | 95.7 (78.1-99.9) | 54.2 (32.8-74.5) |
| PAI-1(total) | 348736.6 (261199.3-456794.4) | 246289.2 (175941.2-350988.5) | 0.018694 | 0.70 (0.55-0.85) | >255621.0 | 78.3 (56.3-92.5) | 58.3 (36.6-77.9) |
| PDGF-AB/BB | 49576.6 (33649.3-83528.9) | 33592.0 (14786.0-49751.6) | 0.032444 | 0.68 (0.53-0.84) | >42307.0 | 65.2 (42.7-83.6) | 66.7 (44.7-84.4) |
| #MBL | 9533.4 (4686.1-30439.6) | 3299.1 (901.1-14882.4) | 0.033928 | 0.68 (0.52-0.84) | >4522.0 | 78.3 (56.3-92.5) | 58.3 (36.6-77.9) |
| NCAM-1 | 246692.5 (164329.5-305706.5) | 285446.4 (256271.6-342048.0) | 0.036064 | 0.68 (0.52-0.84) | <264419.0 | 69.6 (47.1-86.8) | 70.8 (48.9-87.4) |
| #CC4b | 29843.2 (21128.5-42752.7) | 25562.6 (17752.8-31264.4) | 0.056822 | 0.66 (0.51-0.82) | >26285.0 | 69.6 (47.1-86.8) | 54.2 (32.8-74.5) |
| MMP-1 | 5694.6 (3233.2-7609.0) | 4084.8 (2174.5-6345.7) | 0.068827 | 0.66 (0.50-0.81) | >4282.0 | 60.9 (38.5-80.3) | 54.2 (32.8-74.5) |
| IL-17A | 0.0 (0.0-0.0) | 0.0 (0.0-18.4) | 0.018640 | 0.65 (0.53-0.76) | <11.3 | 95.7 (78.1-99.9) | 37.5 (18.8-59.4) |
| CXCL8/IL-8 | 37.1 (15.5-54.1) | 55.4 (27.5-112.9) | 0.072101 | 0.65 (0.49-0.81) | <42.1 | 60.9 (38.5-80.3) | 66.7 (44.7-84.4) |
| #CC4 | 157528.9 (90929.3-209684.1) | 85388.5 (48405.5-194821.2) | 0.079129 | 0.65 (0.49-0.81) | >89484.0 | 78.3 (56.3-92.5) | 54.2 (32.8-74.5) |
| sRAGE | 855.2 (773.7-896.6) | 875.8 (855.2-937.8) | 0.094181 | 0.64 (0.48-0.80) | <875.8 | 73.9 (51.6-89.8) | 50.0 (29.1-70.9) |
| TGF-α | 60.3 (26.9-96.2) | 28.5 (5.6-79.8) | 0.110002 | 0.64 (0.48-0.80) | >29.9 | 69.6 (47.1-86.8) | 54.2 (32.8-74.5) |
| IL-7 | 36.0 (22.9-55.8) | 29.2 (12.4-37.7) | 0.110363 | 0.64 (0.48-0.80) | >27.5 | 69.6 (47.1-86.8) | 50.0 (29.1-70.9) |
| IL-6 | 6.8 (1.6-14.6) | 8.9 (2.5-44.7) | 0.135692 | 0.63 (0.47-0.79) | <8.0 | 56.5 (34.5-76.8) | 58.3 (36.6-77.9) |
| #Apo AI | 302283.6 (267898.0-346446.2) | 286350.3 (191698.6-320139.9) | 0.160089 | 0.62 (0.46-0.78) | >287512.0 | 65.2 (42.7-83.6) | 54.2 (32.8-74.5) |
| VEGF A | 152.4 (112.5-251.2) | 106.7 (74.8-235.8) | 0.169862 | 0.62 (0.45-0.78) | >111.2 | 78.3 (56.3-92.5) | 54.2 (32.8-74.5) |
| #CF1 | 66236.8 (49972.0-99204.5) | 54181.1 (45646.3-71882.7)) | 0.176578 | 0.62 (0.45-0.78) | >57835.0 | 65.2 (42.7-83.6) | 62.5 (40.6-81.2) |
| MMP-7 | 808.0 (524.4-1584.1) | 1175.0 (625.5-3399.8) | 0.189921 | 0.61 (0.45-0.78) | <869.0 | 60.9 (38.5-80.3) | 62.5 (40.6-81.2) |
| #Myoglobin | 9.6 (4.4-20.3) | 21.4 (4.9-51.0) | 0.201135 | 0.61 (0.44-0.78) | <10.2 | 60.9 (38.5-80.3) | 66.7 (44.7-84.4) |
| CXCL10/IP-10 | 55.9 (35.9-169.1) | 75.8 (49.3-298.3) | 0.213146 | 0.61 (0.44-0.77) | <57.2 | 52.2 (30.6-73.2) | 66.7 (44.7-84.4) |
| PDGF-AA | 8538.7 (5683.1-15788.5) | 6995.0 (2635.5-12806.3) | 0.221073 | 0.61 (0.44-0.77) | >6150.0 | 69.6 (47.1-86.8) | 50.0 (29.1-70.9) |
| #MIP4 | 241.6 (172.5-366.9) | 178.1 (119.2-342.3) | 0.221073 | 0.61 (0.44-0.77) | >187.7 | 69.6 (47.1-86.8) | 54.2(32.8-74.5) |
| #A1AT | 18729.1 (14631.0-24621.2) | 16819.0 (11711.3-27780.9) | 0.287284 | 0.59 (0.42-0.76) | >17908.0 | 60.9 (38.5-80.3) | 58.3 (36.6-77.9) |
| MMP-8 | 24763.5 (12747.3-86623.8) | 19342.7 (9257.2-35601.6) | 0.360001 | 0.59 (0.41-0.75) | >22769.0 | 56.5 (34.5-76.8) | 58.3 (36.6-77.9) |
| Aβ42 | 0.0 (0.0-0.0) | 0.0 (0.0-556.9) | 0.240593 | 0.58 (0.45-0.72) | <278.4 | 73.9 (51.6-89.8) | 41.7 (22.1-63.4) |
| #P-Selectin | 194.3 (102.1-352.1) | 119.1 (54.4-274.0) | 0.330420 | 0.58 (0.42-0.75) | >159.1 | 65.2 (42.7-83.6) | 62.5 (40.6-81.2) |
| CC5a | 2663.1 (1751.2-3946.9) | 2423.2 (1559.1-3554.3) | 0.349063 | 0.58 (0.41-0.75) | >2660.0 | 52.2 (30.6-73.2) | 66.7 (44.7-84.4) |
| CCL3/MIP-1β | 48.7 (0.0-65.1) | 49.8 (0.0-209.1) | 0.382647 | 0.57 (0.41-0.74) | <48.9 | 65.2 (42.7-83.6) | 54.2 (32.8-74.5) |
| MMP-9 | 205449.19 (59802.48-556493.88) | 174486.7 (73396.4-266465.9) | 0.387093 | 0.57 (0.40-0.74) | >189764.0 | 56.5 (34.5-76.8) | 58.3 (36.6-77.9) |
| Cathepsin D | 439708.5 (308272.3-728466.0) | 493856.4 (331662.9-959098.3) | 0.412583 | 0.57 (0.40-0.74) | <459422.0 | 60.9 (38.5-80.3) | 54.2 (32.8-74.5) |
| GM-CSF | 0.0 (0.0-0.0) | 0.0 (0.0-0.0) | 0.158099 | 0.57 (0.40-0.73) | <9.3 | 100.0 (85.2-100) | 16.7 (4.7-37.4) |
| ICAM-1 | 216547.6 (137559.5-286618.4) | 215566.5 (171273.1-337326.2) | 0.418679 | 0.57 (0.40-0.72) | <224039.0 | 56.5 (34.5-76.8) | 50.0 (29.1-70.9) |
| #CRP | 230000.0 (230000.0-230000.0) | 230000.0 (63731.2-230000.0) | 0.380342 | 0.56 (0.43-0.69) | >80721.0 | 87.0 (66.4-97.2) | 33.3 (15.6-55.3) |
| IL-1β | 0.0 (0.0-0.0) | 0.0 (0.0-9.2) | 0.358788 | 0.56 (0.43-0.68) | <8.3 | 91.3 (72.0-98.9) | 29.2 (12.6-51.1) |
| #CC9 | 3295.9 (2497.1-4084.6) | 3657.9 (2600.8-4489.9) | 0.475866 | 0.56 (0.39-0.73) | <3502.0 | 65.2 (42.7-83.6) | 58.3 (36.6-77.9) |
| MPO | 4746700.0 (1779300.0-6026200.0) | 3438600.0 (1669250.0-4934150.0) | 0.475891 | 0.56 (0.39-0.73) | >4650000.0 | 52.2 (30.6-73.2) | 70.8 (48.9-87.4) |
| CD40L | 11633.0 (8228.9-16525.6) | 10742.2 (6930.7-17042.3) | 0.475891 | 0.56 (0.39-0.73) | >11151.0 | 65.2 (42.7-83.6) | 54.2 (32.8-74.5) |
| #GDF-15 | 1.0 (0.6-1.6) | 1.1 (0.6-3.1) | 0.501871 | 0.56 (0.39-0.73) | <1.1 | 60.9 (38.5-80.3) | 54.2 (32.8-74.5) |
| IL-21 | 0.0 (0.0-0.0) | 0.0 (0.0-15.9) | 0.396732 | 0.55 (0.43-0.67) | <34.6 | 95.7 (78.1-99.9) | 20.8 (7.1-42.2) |
| #D-dimer | 9287.6 (1772.3-17900.1) | 9102.9 (3021.2-41007.9) | 0.550286 | 0.55 (0.38-0.72) | <9451.0 | 52.2 (30.6-73.2) | 50.0 (29.1-70.9) |
| BDNF | 15636.7 (10109.5-24406.5) | 18107.0 (8952.6-28946.9) | 0.572783 | 0.55 (0.38-0.72) | <17211.0 | 65.2 (42.7-83.6) | 54.2 (32.8-74.5) |
| CXCL9/MIG | 2309.5 (0.0-3311.4) | 1800.7 (0.0-3557.3) | 0.625319 | 0.54 (0.38-0.71) | >2114.0 | 52.2 (30.6-73.2) | 62.5 (40.6-81.2) |
| Aβ40 | 0.0 (0.0-0.0) | 0.0 (0.0-0.0) | 0.171006 | 0.54 (0.37-0.71) | <72.1 | 100.0 (85.2-100.0) | 8.3 (1.0-27.0) |
| #SAA | 65700.0 (847.0-230000.0) | 39439.7 (6551.9-226031.8) | 0.656243 | 0.54 (0.37-0.71) | >59894.0 | 56.5 (34.5-76.8) | 66.7 (44.7-84.4) |
| IL-13 | 0.0 (0.0-338.1) | 0.0 (0.0-756.3) | 0.681743 | 0.53 (0.38-0.69) | <74.6 | 56.5 (34.5-76.8) | 45.8 (25.6-67.2) |
| #CC2 | 15903.9 (8706.0-31171.1) | 15768.9 (6992.3-49343.7) | 0.725481 | 0.53 (0.36-0.70) | <15990.0 | 52.2 (30.6-73.2) | 50.0 (29.1-70.9) |
| Ferritin | 52841.0 (14202.0-114067.7) | 62740.4 (16776.2-169542.0) | 0.740490 | 0.53 (0.36-0.70) | <56314.0 | 56.5 (34.5-76.8) | 58.3 (36.6-77.9) |
| #PEDF | 21756.5 (18654.6-25542.3) | 21401.6 (18159.9-26348.1) | 0.765743 | 0.53 (0.36-0.70) | >21725.0 | 52.2 (30.6-73.2) | 54.2 (32.8-74.5) |
| IL-12/23p40 | 0.0 (0.0-0.0) | 0.0 (0.0-0.0) | 0.349077 | 0.52 (0.35-0.69) | <620.1 | 100.0 (85.2-100.0) | 4.2 (0.1-21.1) |
| RANTES | 108231.8 (53485.5-169473.6) | 92692.2 (39285.6-188178.1) | 0.790226 | 0.52 (0.35-0.69) | >99016.0 | 56.5 (34.5-76.8) | 54.2 (32.8-74.5) |
| #NGAL | 394.1 (152.5-1046.1) | 380.5 (189.3-560.3) | 0.831299 | 0.52 (0.35-0.69) | >371.5 | 52.2 (30.6-73.2) | 50.0 (29.1-70.9) |
| CCL1/I-309 | 15.0 (8.6-33.4) | 15.2 (7.6-44.4) | 0.848035 | 0.52 (0.35-0.69) | <15.2 | 52.2 (30.6-73.2) | 50.0 (29.1-70.9) |
| #ADMTS13 | 901.2 (545.3-1092.7) | 874.4 (600.0-1120.0) | 0.823096 | 0.52 (0.35-0.68) | <962.3 | 60.9 (38.5-80.3) | 45.8 (25.6-67.2) |
| IFN-γ | 0.0 (0.0-0.0) | 0.0 (0.0-0.0) | 0.928917 | 0.51 (0.39-0.63) | <61.5 | 87.0 (66.4-92.2) | 20.8 (7.1-42.2) |
| GDNF | 136.3 (120.1-152.7) | 136.3 (136.3-152.7) | 0.921886 | 0.51 (0.34-0.67) | <140.4 | 52.2 (30.6-73.2) | 41.7 (22.1-63.4) |
| S100B | 2800.0 (2744.2-2800.0) | 2800.0 (2744.2-2800.0) | 0.986591 | 0.50 (0.34-0.66) | >2772.0 | 55.6 (30.8-78.5) | 40.0 (19.1-64.0) |
| *Cathelicidin-LL37 | 0.5 (0.3-0.9) | 0.5 (0.3-0.9) | 0.974533 | 0.49 (0.31-0.66) | >0.4 | 60.9 (38.5-80.3) | 34.8 (16.4-57.3) |
| #CC3 | 40885.9 (36448.0-74127.5) | 46059.4 (25390.4-53871.9) | 0.254876 | 0.40 (0.23-0.57) | >32056.0 | 91.3 (72.0-98.9) | 41.7 (22.1-63.4) |

1. Manyelo CM, Solomons RS, Snyders CI, Manngo PM, Mutavhatsindi H, Kriel B, Stanley K, Walzl G, Chegou NN. Application of Cerebrospinal Fluid Host Protein Biosignatures in the Diagnosis of Tuberculous Meningitis in Children from a High Burden Setting. *Mediators Inflamm* (2019) doi:10.1155/2019/7582948
