# Supplementary material for: Potential of Host Serum Protein Biomarkers in the Diagnosis of Tuberculous Meningitis in Children
Source: Front Pediatr. 2019 Sep 25;7:376. doi: 10.3389/fped.2019.00376 (PMC6773834; doi:10.3389/fped.2019.00376)
Supplement: Supplementary file 2 [file Table_2.DOCX]

**Supplementary Table 2.** **Proportion of study participants with host markers above the minimum detectable concentration (MDC) in CSF and serum samples and the differences in median levels between the two sample types.** MDC values were obtained from the package inserts provided by the kit manufacturers. *Values shown are the optical density (OD). #Marker levels are in ng/ml, while the other markers are in pg/ml. IQR: Inter-quartile range. The performance of the biomarkers in diagnosing TBM, when measured in CSF samples has been reported previously in (1). P-values for differences in expression of the different biomarkers in cerebrospinal fluid (1) and serum were calculated using the Mann Whitney U test.

|  | **Cerebrospinal fluid (n=47)** | | | **Serum (n=47)** | |  | |
| --- | --- | --- | --- | --- | --- | --- | --- |
| **Marker** | **MDC** | **% > MDC** | **Median (IQR), (n=47)** | **% > MDC** | **Median (IQR), (n=47)** | ***P* value (CSF vs serum)** | |
| (A) Host markers more abundantly expressed in CSF | | | | | | | |
| Aβ40 | 1,4 | 97.9 | 647.6 (305.1-1407.0) | 4.3 | 0.0 (0.0-0.0) | <0.0001 | |
| Aβ42 | 9,7 | 93.6 | 200.6 (54.3-409.4) | 34.0 | 0.0 (0.0-556.9) | 0.0008 | |
| CCL3/MIP-1α | 101.2 | 78.7 | 219.1 (118.9-327.5) | 27.7 | 48.7 (0.0-180.4) | <0.0001 | |
| CXCL8/IL-8 | 5.2 | 100 | 454.9 (107.8-1106.0) | 93.6 | 46.6 (20.4-78.0) | <0.0001 | |
| IFN-γ | 58.5 | 57.4 | 91.7 (7.7-469.9) | 14.9 | 0.0 (0.0-0.0) | <0.0001 | |
| IL-10 | 4.8 | 74.5 | 16.0 (3.9-50.4) | 38.3 | 1.3 (0.0-8.8) | <0.0001 | |
| IL-13 | 455 | 42.6 | 378.2 (169.1-1171.0) | 25.5 | 0.0 (0.0-756.3) | 0.0005 | |
| IL-21 | 29.4 | 74.5 | 44.7 (28.6-71.0) | 14.9 | 0.0 (0.0-0.0) | <0.0001 | |
| IL-6 | 4.8 | 72.3 | 86.8 (2.3-536.6) | 61.7 | 8.9 (1.8-25.9) | 0.0017 | |
| CCL2/MCP-1 | 33 | 100 | 993.4 (502.2-1395.0) | 100 | 329.1 (228.9-640.0) | <0.0001 | |
| CXCL10/IP-10 | 2.8 | 100 | 1974.0 (189.8-44900.0) | 100 | 66.4 (39.3-195.4) | <0.0001 | |
| CXCL9/MIG | 586 | 97.9 | 3163.0 (1350.0-9846.0) | 68.1 | 1801.0 (0.0-3311.0) | 0.0021 | |
| GM-CSF | 12.2 | 89.4 | 63.0 (20.7-93.0) | 8.5 | 0.0 (0.0-0.0) | <0.0001 | |
| IL-1β | 19.5 | 48.9 | 13.9 (0.0-51.3) | 10.6 | 0.0 (0.0-0.0) | <0.0001 | |
| IL-12/23p40 | 383 | 27.7 | 0.0 (0.0-542.3) | 2.1 | 0.0 (0.0-0.0) | <0.0001 | |
| IL-17A | 12.8 | 36.2 | 9.2 (0.0-20.5) | 19.1 | 0.0 (0.0-0.0) | 0.001 | |
| IL-4 | 14.6 | 100 | 170.2 (113.3-246.1) | 87.2 | 99.6 (41.1-136.8) | <0.0001 | |
| TNF-α | 9.7 | 57.4 | 22.1 (1.2-71.4) | 57.4 | 14.4 (0.0-24.9) | 0.0736 | |
| (B) Host markers more abundantly expressed in serum | | | | | | | |
| #D-dimer | 0.028 | 100.0 | 1448 (2.5-98000.0) | 100.0 | 9283.0 (2437.0-21283.0) | | 0.7202 |
| #CC2 | 0.25 | 100.0 | 774.4 (70.8-2297.0) | 100.0 | 15904.0 (7911.0-37262.0) | | <0.0001 |
| #CC4b | 0.28 | 100.0 | 351.9 (168.7-611.0) | 100.0 | 28264.0 (19539.0-35483.0) | | <0.0001 |
| #CC5 | 0.68 | 93.6 | 166.1 (33.3-511.3) | 100.0 | 46216.0 (34488.0-55936.0) | | <0.0001 |
| CC5a | 0.0023 | 100.0 | 41.3 (6.4-88.0) | 100.0 | 2530.0 (1645.0-3761.0) | | <0.0001 |
| #CC9 | 8.16 | 93.6 | 35.7 (24.6-50.0) | 100.0 | 3423.0 (2584.0-4293.0) | | <0.0001 |
| #Adipsin/CFD | 0.016 | 100.0 | 43.5 (21.2-119.4) | 100.0 | 2468.0 (1791.0-3458.0) | | <0.0001 |
| #MBL | 0.036 | 93.6 | 5.3 (0.9-25.6) | 100.0 | 7544.0 (1636.0-18866.0) | | <0.0001 |
| #CF1 | 0.15 | 100.0 | 275.7 (97.6-699.5) | 100.0 | 59466.0 (45700.0-77858.0) | | <0.0001 |
| #P-selectin | 0.024 | 44.7 | 0.0-0.0-1.6) | 78.7 | 166.5 (60.7-291.1) | | <0.0001 |
| VCAM-1 | 0.032 | 80,9 | 27.9 (6.8-129.4) | 80.9 | 957.1 (631.5-1408.0) | | <0.0001 |
| #ADAMTS13 | 0.053 | 100.0 | 7.2 (0.6-13.3) | 100.0 | 891.5 (545.3-1093.0) | | <0.0001 |
| #GDF-15 | 0.00011 | 80.9 | 0.2 (0.0-0.4) | 95.7 | 1.0 (0.6-2.1) | | <0.0001 |
| #Myoglobin | 0.007 | 80.9 | 0.2 (0.0-1.0) | 100.0 | 11.1 (4.7-37.8) | | <0.0001 |
| #Lipocalin-2/NGAL | 0.001 | 80.9 | 7.9 (0.8-78.4) | 87.2 | 394.1 (170.6-691.6) | | 0.0004 |
| #SAA | 0.048 | 76.6 | 52.3 (0.2-2411.0) | 80.9 | 46890.0 (4510.0-230000.0) | | 0.0018 |
| BDNF | 0.23 | 63.8 | 0.6 (0.0-1.0) | 100.0 | 16046.0 (10109.0-25813.0) | | <0.0001 |
| Cathepsin D | 8.08 | 100.0 | 73531.0 (55896.0-95805.0) | 100.0 | 450337.0 (315790.0-746949.0) | | <0.0001 |
| ICAM-1 | 6.29 | 100.0 | 1503.0 (420.1-3384.0) | 100.0 | 216548.0 (148077.0-303985.0) | | <0.0001 |
| MPO | 200.0 | 89.4 | 28780.0 (1383.0-63392.0) | 100.0 | 3770000.0 (1690000.0-5750000.0) | | <0.0001 |
| PDGF-AA | 0.22 | 95.7 | 7.3 (5.0-15.9) | 100.0 | 8089.0 (3553.0-14143.0) | | <0.0001 |
| RANTES | 1.20 | 83.0 | 9.3 (3.7-22.3) | 100.0 | 102077.0 (49156.0-185835.0) | | <0.0001 |
| PDGF-AB/BB | 3.83 | 76.6 | 7.0 (4.1-12.9) | 100.0 | 41696.0 (20984.0-69370.0) | | <0.0001 |
| PAI-1 | 0.48 | 100.0 | 1706.0 (348.2-9135.0) | 100.0 | 271962.0 (198549.0-387515.0) | | <0.0001 |
| S100B | 3.1 | 80,9 | 41.2 (30.1-766.1) | 80.9 | 2800.0 (2744.0-2800.0) | | <0.0001 |
| sRAGE | 3,8 | 97.9 | 14.1 (12.8-16.0) | 100.0 | 855.2 (773.7-937.8) | | <0.0001 |
| GDNF | 0,9 | 80,9 | 2.1 (1.8-2.3) | 83.0 | 136.3 (120.1-152.7) | | <0.0001 |
| Ferritin | 25.8 | 100 | 3261.0 (621.2-8447.0) | 80.9 | 57058,0 (15940,0-137448,0) | | 0.0012 |
| MMP-9 | 134.1 | 61.7 | 1480.0 (2.6-4385.0) | 80.9 | 188899.0 (59802.0-348674.0) | | <0.0001 |
| CD40L | 212 | 76.6 | 363.4 (214.6-594.4) | 100 | 11509.0 (7489.0-16633.0) | | <0.0001 |
| MMP-1 | 50 | 100 | 398.5 (280.1-770.2) | 100 | 4363.0 (2518.0-7221.0) | | <0.0001 |
| TGF-α | 18.6 | 19.1 | 7.3 (2.2-14.8) | 76.6 | 43.0 (22.6-91.9) | | <0.0001 |
| IL-7 | 4.5 | 51.1 | 5.0 (1.6-7.0) | 97.9 | 31.6 (14.8-46.3) | | <0.0001 |
| MMP-8 | 245.1 | 72.3 | 1985.0 (60.0-8791.0) | 89.4 | 21071.0 (11071.0-45371.0) | | <0.0001 |
| #Apo AI | 0.300 | 85.1 | 980.1 (150.8-4219.0) | 100.0 | 295552.0 (239391.0-324560.0) | | <0.0001 |
| #Apo CIII | 0.001 | 100.0 | 29.4 (11.0-167.2) | 100.0 | 134435.0 (86406.0-167953.0) | | <0.0001 |
| #CC3 | 0.012 | 100.0 | 657.6 (154.2-1449.0) | 100.0 | 44827.0 (31909.0-67779.0) | | <0.0001 |
| #CFH | 0.037 | 91.5 | 746.1 (200.7-2276.0) | 100.0 | 374790.0 (293798.0-447723.0) | | <0.0001 |
| #CRP | 0.0022 | 100.0 | 2387.0 (239.5-230000.0) | 100.0 | 230000.0 (92176.0-230000.0) | | 0.0003 |
| #A1AT (α1-Antitrypsin) | 0.0362 | 100.0 | 916.0 (319.1-2721.0) | 100.0 | 18344.0 (13298.0-25608.0) | | <0.0001 |
| #PEDF | 0.008 | 100.0 | 735.7 (606.7-831.0) | 100.0 | 21693.0 (18259.0-25919.0) | | <0.0001 |
| #SAP | 0.009 | 100.0 | 34.6 (8.8-121.3) | 100.0 | 269193.0 (140485.0-491860.0) | | <0.0001 |
| #MIP-4 | 0.0041 | 100.0 | 5.4 (0.3-47.5) | 100.0 | 223.7 (146.9-366.9) | | <0.0001 |
| #CC4 | 0.0465 | 97.9 | 639.6 (297.1-1473.0) | 95.7 | 146270.0 (70465.0-204543.0) | | <0.0001 |
| MMP-7 | 350 | 8.5 | 101.5 (81.6-121.5) | 87.2 | 930.0 (605.2-1748.0) | | <0.0001 |
| VEGF-A | 8.4 | 55.3 | 10.0 (2.8-78.4) | 100 | 142.8 (77.1-241.8) | | <0.0001 |
| NCAM1 | 872 | 100 | 115167.0 (59829.0-172190.0) | 100 | 265229.0 (209949.0-339134.0) | | <0.0001 |
| *Cathelicidin-LL37 | n/a | n/a | 0.0 (0.0-0.0) | n/a | 0.5 (0.3-0.9) | | <0.0001 |
| (C) Host markers showing no difference in expression in CSF and serum samples | | | | | | | |
| CCL4 | 150.2 | 74.5 | 240.6 (148.4-470.0) | 87.2 | 296.8 (179.8-431.0) | | 0.4363 |
| G-CSF | 24 | 63.8 | 173.4 (0.0-458.7) | 61.7 | 83.0 (0.0-162.9) | | 0.1172 |
| CCL1/I-309 | 4.57 | 78.7 | 63.2 (5.13-156.6) | 91.5 | 15.0 (7.6-33.4) | | 0.13 |

Abbreviations:

MDC : minimum detectable concentration, OD : optical density, n/a : not applicable (OD was reported), IQR : interquartile range, CSF : cerebrospinal fluid, TBM : tuberculous meningitis.

**References:**

1. Manyelo CM, Solomons RS, Snyders CI, Manngo PM, Mutavhatsindi H, Kriel B, Stanley K, Walzl G, Chegou NN. Application of Cerebrospinal Fluid Host Protein Biosignatures in the Diagnosis of Tuberculous Meningitis in Children from a High Burden Setting. *Mediators Inflamm* (2019) doi:10.1155/2019/7582948
